# Free Fatty Acids in Bone Pathophysiology of Rheumatic Diseases

**DOI:** 10.3389/fimmu.2019.02757

**Published:** 2019-12-03

**Authors:** Klaus W. Frommer, Rebecca Hasseli, Andreas Schäffler, Uwe Lange, Stefan Rehart, Jürgen Steinmeyer, Markus Rickert, Kerstin Sarter, Mario M. Zaiss, Carsten Culmsee, Goutham Ganjam, Susanne Michels, Ulf Müller-Ladner, Elena Neumann

**Affiliations:** ^1^Department of Rheumatology and Clinical Immunology, Justus-Liebig-University Gießen, Giessen, Germany; ^2^Department of Internal Medicine III, Endocrinology, Diabetes, Metabolism, Justus-Liebig-University of Giessen, Giessen, Germany; ^3^Department of Orthopedics and Trauma Surgery, Agaplesion Markus Hospital, Frankfurt, Germany; ^4^Department of Orthopaedics and Orthopaedic Surgery, University Hospital Giessen and Marburg, Giessen, Germany; ^5^Department of Internal Medicine 3, Rheumatology and Immunology, Friedrich-Alexander-University Erlangen-Nürnberg (FAU) and Universitätsklinikum Erlangen, Erlangen, Germany; ^6^Institute for Pharmacology and Clinical Pharmacy, University of Marburg, Marburg, Germany; ^7^Center for Mind Brain and Behavior (CMBB), Universities of Marburg and Gießen, Marburg, Germany; ^8^Department of Neurology, Philipps University Marburg, Marburg, Germany

**Keywords:** fatty acid, inflammation, osteoblasts, osteoclasts, rheumatoid arthritis, osteoarthritis

## Abstract

Obesity—in which free fatty acid (FFA) levels are chronically elevated—is a known risk factor for different rheumatic diseases, and obese patients are more likely to develop osteoarthritis (OA) also in non-weight-bearing joints. These findings suggest that FFA may also play a role in inflammation-related joint damage and bone loss in rheumatoid arthritis (RA) and OA. Therefore, the objective of this study was to analyze if and how FFA influence cells of bone metabolism in rheumatic diseases. When stimulated with FFA, osteoblasts from RA and OA patients secreted higher amounts of the proinflammatory cytokine interleukin (IL)-6 and the chemokines IL-8, growth-related oncogene α, and monocyte chemotactic protein 1. Receptor activator of nuclear factor kappa B ligand (RANKL), osteoprotegerin, and osteoblast differentiation markers were not influenced by FFA. Mineralization activity of osteoblasts correlated inversely with the level of FFA-induced IL-6 secretion. Expression of the Wnt signaling molecules, axin-2 and β-catenin, was not changed by palmitic acid (PA) or linoleic acid (LA), suggesting no involvement of the Wnt signaling pathway in FFA signaling for osteoblasts. On the other hand, Toll-like receptor 4 blockade significantly reduced PA-induced IL-8 secretion by osteoblasts, while blocking Toll-like receptor 2 had no effect. In osteoclasts, IL-8 secretion was enhanced by PA and LA particularly at the earliest time point of differentiation. Differences were observed between the responses of RA and OA osteoclasts. FFA might therefore represent a new molecular factor by which adipose tissue contributes to subchondral bone damage in RA and OA. In this context, their mechanisms of action appear to be dependent on inflammation and innate immune system rather than Wnt-RANKL pathways.

## Introduction

Obesity is not just a matter of excess body weight, but it is also associated with numerous inflammatory cardiovascular and metabolic diseases such as atherosclerosis, coronary heart disease, and type 2 diabetes (1). This link to inflammation can be attributed to adipose tissue being a source of various biologically active mediators including cytokines [e.g., tumor necrosis factor, interleukin (IL)-6] (2–4), adipokines (e.g., adiponectin, visfatin) (5, 6), complement factors (7, 8), and certain free fatty acids (FFA) (9). These factors may in part be responsible for the long-term comorbidities of obesity (10–12). In particular, serum/plasma levels of FFA, i.e., fatty acids, which are not covalently bound to other molecules, are chronically elevated in obese individuals compared to lean individuals (9, 13, 14). Results from *in vitro* experiments, in which FFA modulated the gene expression of adipocytes (15) and hepatocytes (16), suggest that they may contribute to cardiovascular and metabolic diseases. However, they may also be involved in rheumatic diseases. Obesity is a known risk factor for different rheumatic diseases (17–21) including osteoarthritis (OA) and rheumatoid arthritis (RA). Several observations support the notion that this is not merely due to increased mechanical stress. For instance, obesity not only causes a higher incidence of arthritis in weight-bearing joints but also in non-weight-bearing joints such as the hands (17, 22–24). It has also been shown that body fat is more detrimental in OA than excess body weight since changes in body fat rather than body weight were related to the symptomatic relief of obese patients with OA (25). Notably, this was not due to increased muscle strength or improved knee-joint alignment as neither of these were associated with the degree of symptomatic relief (26). Several animal models support the role of obesity or a high-fat diet in OA: In mice, high fat diet-induced obesity caused OA and systemic inflammation in proportion to body fat, while OA symptoms were not deteriorated but instead alleviated by increased mechanical joint loading via intense long-term exercise (27). Surgically induced OA in mice was accelerated by short- and long-term high fat diets (28), and obese mice developed more severe OA caused by intra-articular fracture than control mice (29). A possible link between metabolic factors and OA is also suggested by the observation that the subtype of metabolic OA sets in earlier and progresses more quickly in comparison to other subtypes while at the same time being accompanied by chronic low grade inflammation (30). Interestingly, a recent study showed increased FFA serum levels in RA patients and in individuals at risk for RA (31). This is in line with our previous findings showing proinflammatory effects of FFA on RA synovial fibroblasts, endothelial cells, and chondrocytes (32). However, the joint pathology in OA and RA also includes the subchondral bone (33, 34), exhibiting hypomineralization and/or changes in microstructure. A potential pathophysiological role of FFA in osteoporosis is also suggested by clinical studies showing associations between the relative proportion of bone marrow adipose tissue, another distinct fat depot, and bone mineral density (35, 36) and animal studies showing a negative effect of high-fat diets on bone density (37, 38). In this study, we therefore investigated whether selected FFA affect cells of bone remodeling, specifically palmitic acid, a saturated fatty acid, and linoleic acid, an unsaturated omega-6 fatty acid, which are the two most abundant FFA in plasma (39).

## Materials and Methods

### Isolation and Culture of Murine Primary Osteoblasts

Calvariae of female 4-day-old C57BL/6J mice were used for obtaining primary murine osteoblasts. For each of the six experiments (*n* = 6), calvariae from five mice were sequentially digested in 1 ml alpha-Minimal Essential Media (α-MEM) (Gibco/Invitrogen) containing 0.1% collagenase type IA (Sigma-Aldrich) and 0.2% dispase II (Sigma-Aldrich) while shaking for 10 min per fraction at 37°C. Cells isolated from the fractions were combined as osteoblastic cells and expanded for 6 days. For differentiation, osteoblasts were cultured in α-MEM (Gibco) supplemented with 10% fetal bovine serum (FBS) (Biochrom) and 1% penicillin/streptomycin (Invitrogen) for 21 days.

### Human Bone Tissue and Blood

Bone tissues were obtained from RA and OA patients who were undergoing knee-joint replacement surgery. Blood samples were obtained from RA and OA patients during regular medical examinations. The patient characteristics are listed in Tables 1, 2. The study was approved by the local Ethics Committee, and all patients gave written informed consent before tissue collection or blood donation. All specimens were taken from patients fulfilling the criteria of the American College of Rheumatology for RA (40) or OA (41).

**Table 1:** Characteristics of rheumatoid arthritis (RA) and osteoarthritis (OA) patients donating bone samples.

	**RA patients**	**OA patients**
*n*	15	20
% female	93%	70%
% male	7%	30%
Age mean ± SD	70 ± 8	62 ± 14
Age median (IQR)	62 (49−75)	71 (65−76)
Age range	43–79	56–85
MTX treatment (Y/N/–)	6/9/–	1/15/4
Corticosteroid treatment (Y/N/–)	13/2/–	1/15/4
Biologics treatment (Y/N/–)	7/8/–	0/16/4

*SD, standard deviation; IQR, interquartile range; MTX, methotrexate; Y/N/–, yes/no/information not available*.

**Table 2:** Characteristics of rheumatoid arthritis (RA) and osteoarthritis (OA) patients donating blood.

	**RA patients**	**OA patients**
*n*	4	4
% female	25%	100%
% male	75%	0%
Age mean ± SD	67 ± 10	67 ± 3
Age median (IQR)	71 (65−74)	68 (66−69)
Age range	52–75	63–70

*SD, standard deviation; IQR, interquartile range*.

### Isolation and Culture of Human Primary Osteoblasts

Samples of cancellous bone tissue were cut into pieces of ~2 mm^2^ in size and washed with phosphate-buffered saline (Biochrom). The bone fragments were incubated with a 1:1 mixture of trypsin/ethylenediaminetetraacetic acid (Capricon Scientific) and phosphate-buffered saline for 5 min at 37°C. Predigestion was stopped by adding the osteoblast culture medium MEM + GlutaMAX™ (Gibco/Invitrogen) supplemented with 10% FBS (Sigma-Aldrich), 100 U/ml penicillin and 10 μg/ml streptomycin (both AppliChem). The supernatant was carefully removed, and the bone fragments were resuspended in fresh culture medium and transferred into 75-cm^2^ culture flasks to allow for cellular outgrowth. After reaching confluency, cells were detached using trypsin/ethylenediaminetetraacetic acid and used for further culturing up to passage 2. Cells were cultured at 37°C and 5% CO_2_.

### Stimulation of Osteoblasts With Free Fatty Acids

Murine osteoblasts and human osteoblasts (in passage 2) were stimulated with FFA for 24 h. Cells were grown to 100% confluency before stimulation. FFA were obtained from Sigma-Aldrich at the highest purity available (chemically synthesized, GC purified). As *in vivo* FFA are mainly bound to serum albumin for increased solubility and prevention of cellular toxicity, fatty acid/bovine serum albumin (BSA) complex solutions were prepared as described previously (32). Fatty acid solutions were sterile filtered before use in stimulation experiments. Two different FFA were used for the stimulation experiments at a concentration of 100 μM: palmitic acid (C16:0) as a saturated fatty acid and linoleic acid (C18:2, n-6) as an unsaturated fatty acid, specifically an omega-6 fatty acid. Controls, i.e., cells to which no FFA were added, were treated with the fatty acid solvent (= vehicle) to exclude effects mediated by the vehicle. The vehicle was prepared in the same manner as the FFA solutions but without adding fatty acids. The equivalent to a 10-mM FFA stock solution consisted of 4.5% (*w*/*v*) BSA and 5% (*v*/*v*) ethanol. Considering the concentration of 100 μM FFA used in the stimulation experiments, this leads to a final concentration of 0.045% (*w*/*v*) BSA and 0.05% (*v*/*v*) ethanol. After stimulation, supernatants were collected for further analysis and cells harvested for RNA isolation. All stimulations were performed in triplicate wells.

### Inhibition of TLR4 and TLR2 Signaling

Toll-like receptor (TLR) 4 and TLR2 were blocked with neutralizing antibodies (5 μg/ml; both from Invivogen). A matched isotype antibody (Invivogen) was used as a control. Cells were preincubated for 2 h with the neutralizing antibodies before stimulation with FFA.

### Mineralization Assay

Primary osteoblasts were seeded into 24-well culture plates at a density of 2 × 10^4^ cells per well and grown to confluency. For the induction of mineralization, the medium was exchanged by osteoblast culture medium supplemented with 5 mM glycerophosphate (Calbiochem) and 100 μg/ml ascorbic acid (Sigma-Aldrich) (= mineralization medium). In parallel, cells were cultured with mineralization medium plus FFA (100 μM) or vehicle to assess the effect of FFA on osteoblast mineralization and with normal osteoblast culture medium as a control. Medium change was performed every 2–3 days (Monday/Wednesday/Friday) until day 21. All stimulations and controls were performed in triplicate wells. After day 21, cells were fixed with formalin (Roth), and mineralized matrix was stained with 2% (*w*/*v*) Alizarin Red S (pH 4.2) (Sigma-Aldrich). Cells were washed with ddH_2_O. The red stain was extracted for quantification using a modified version of the protocol by Gregory et al. (42). For extraction of the stain, 10% (*v*/*v*) acetic acid (Roth) was used, which was neutralized by 10% ammonia solution (Merck). The absorbance was measured at 492 nm.

### Isolation and Culture of Murine Osteoclast Precursors

Bone marrow isolated from three female 8-week-old C57BL/6J mice (*n* = 3) by flushing femoral bones with complete media was used for bone marrow cell isolation. Cells were cultured overnight in α-MEM (Sigma-Aldrich) containing 10% FBS (Sigma-Aldrich) and macrophage colony-stimulating factor (M-CSF) (50 ng/ml) (R&D Systems). After 24 h, non-adherent cells were harvested and seeded in 96-well flat bottom plates (2 × 10^5^ cells per well) in α-MEM supplemented with 10% FBS, 30 ng/ml M-CSF, and 50 ng/ml RANKL (R&D Systems) in the presence or absence of FFA. Medium was changed after 72 h. Cell culture supernatants were collected after 4 days for enzyme-linked immunosorbent assay (ELISA) measurements.

### Isolation of Human Peripheral Blood Mononuclear Cells

Heparinized, peripheral blood was obtained from RA or OA patients after written informed consent, and peripheral blood mononuclear cells (PBMCs) were isolated on a Ficoll 400-based (Biocoll Separating Solution; Biochrom) density gradient. The isolated PBMC were resuspended in MEM + GlutaMAX™ supplemented with 10% FBS, 100 U/ml penicillin + 10 μg/ml streptomycin, and 30 ng/ml M-CSF (Peprotech). For preparation of RNA lysates and collection of supernatants, cells were seeded in 24-well plates at a density of 1.5 × 10^6^ cells per well. Cells were seeded in triplicate wells and cultured at 37°C and 5% CO_2_.

### Differentiation of Human PBMC Into Osteoclasts and Stimulation With Free Fatty Acids

Differentiation of PBMC into osteoclasts was performed as follows: On day 1, the culture medium was exchanged with medium containing 30 ng/ml M-CSF (R&D), 50 ng/ml RANKL (R&D), and 5 ng/ml transforming growth factor beta (R&D). On day 4, concentration of the differentiation factors within the medium was reduced to 10 ng/ml for M-CSF and to 5 ng/ml for RANKL to allow detection of potential effects of FFA on osteoclast differentiation. Starting with day 5, FFA was added to the culture medium, and the medium was changed every 3 days. Cell culture supernatants were collected on days 8, 11, and 14. Cells were harvested for RNA isolation on day 14.

### Real-Time Polymerase Chain Reaction

RNA was isolated from osteoblasts using the RNeasy Mini Kit, according to the manufacturer's instructions (Qiagen). RNA was reverse transcribed into complementary DNA according to a standard protocol using avian myeloblastosis virus reverse transcriptase (Promega) and random hexamer primers (Roche). After denaturation (2 min at 70°C) and immediate cooling down on ice, reverse transcription was performed for 30 min at 42°C, 30 min at 55°C, and 10 min at 70°C. Complementary DNA samples were analyzed by real-time PCR in a LightCycler (Roche) using SYBR Green I. Real-time PCR cycling conditions were 15 min at 95°C, 50 cycles of 15 s at 95°C, 35 s at 53–65°C (depending on the primer pair), and 35 s at 72°C, and were finished using a melting curve analysis program. The reference gene for normalization was 18S ribosomal RNA. The results were analyzed with the Roche LightCycler software and Microsoft Excel according to the ΔΔ*C*_t_ method (43).

### Immunoassays

Protein levels in cell culture supernatants were measured using commercially available ELISAs. Human osteoprotegerin (OPG) was quantified with an ELISA from Enzo Life Sciences and human sRANKL with an ELISA from RayBiotech; all other ELISAs were from R&D Systems.

### Statistical Analysis

Data are presented as arithmetic mean ± standard error of mean (SEM) and were calculated based on biological replicates. Fold changes were log2 transformed before statistical analysis. Normality of data was tested for by the Shapiro–Wilk test. Normally distributed data were analyzed for statistically significant differences by one-way ANOVA + Holm–Sidak's multiple comparisons test, while the Friedman test with Dunn's multiple comparisons test was used for non-normally distributed (paired) data. Correlation analysis was performed according to Pearson. *p* < 0.05 were considered statistically significant. Statistical calculations were performed using GraphPad Prism 6 (GraphPad Software, Inc., La Jolla, USA).

## Results

### Proinflammatory Response of Osteoblasts to Free Fatty Acids

**Figure 1:**
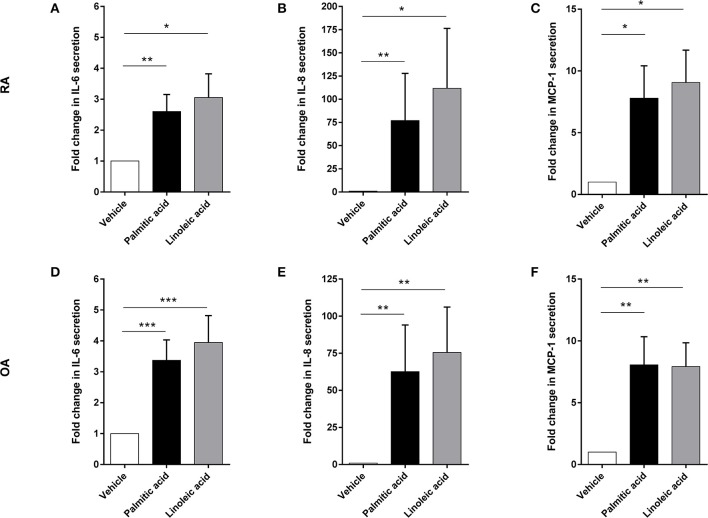
Free fatty acids palmitic acid and linoleic acid induce a proinflammatory response in osteoblasts. Supernatants of RA and OA osteoblasts were analyzed after 24 h stimulation with palmitic acid or linoleic acid for IL-6 (RA: *n* = 14, OA: *n* = 20), IL-8 (RA: *n* = 14, OA: *n* = 20), and MCP-1 (RA: *n* = 3, OA: *n* = 3). Both fatty acids significantly enhanced the secretion of these factors in RA **(A–C)** and OA **(D–F)** osteoblasts. *p < 0.05; **p < 0.001; and ***p < 0.001. MCP-1, monocyte chemotactic protein 1 (CCL2).

**Figure 2:**
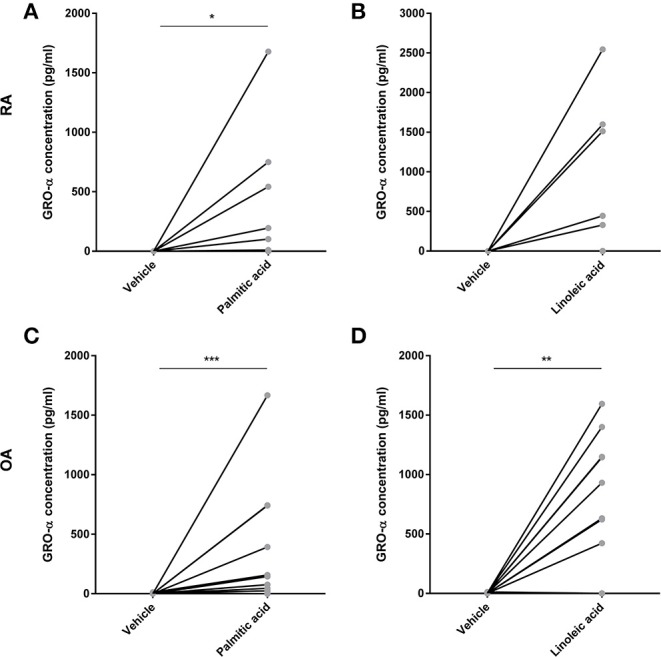
Free fatty acids palmitic acid and linoleic acid induce the secretion of the chemokine GRO-α in osteoblasts. GRO-α was induced from a non-detectable level in RA (**A,B**, *n* = 14) and OA (**C,D**, *n* = 20) in a subset of osteoblast populations. *p < 0.05; **p < 0.001; and ***p < 0.001. GRO-α, Growth-related oncogene α (CXCL1).

Preliminary experiments with murine osteoblasts stimulated with palmitic acid (C16:0) and linoleic acid (C18:2) at 100 μM showed significant increases in IL-6 and monocyte chemotactic protein 1 (MCP-1) secretion (*n* = 6 each) (Figure S1). Hence, RA osteoblasts (*n* = 14) as well as OA osteoblasts (*n* = 20) were stimulated with palmitic acid and linoleic acid at 100 μM. Both fatty acids significantly increased the secretion of the proinflammatory cytokine IL-6 (RA, *n* = 14/OA, *n* = 20) as well as the chemokines IL-8 (RA, *n* = 14/OA, *n* = 20) and MCP-1 (RA, *n* = 3/OA, *n* = 3) by RA and OA osteoblasts (Figure 1). Growth-related oncogene α (GRO-α) secretion was induced from a non-detectable level (<31.2 pg/ml) to a detectable level in OA and RA osteoblasts (Figure 2) in only a subset of human osteoblasts from different patients (5/14 RA osteoblast populations for both FFA; 11/20 OA osteoblast populations for palmitic acid, 8/20 OA osteoblast populations for linoleic acid). However, we did not observe any association of the responses to FFA with the patient data available, which included age, sex, and medication, and there were no significant differences in the responses between RA and OA osteoblasts.

### Effect of Free Fatty Acids on Osteoblast-Expressed Factors Regulating Osteoclastogenesis

Under appropriate conditions, osteoblasts are capable of expressing and secreting the osteoclastogenesis regulating factors “receptor activator of NF-κB ligand” (RANKL) and OPG with OPG being the antagonist of RANKL. Stimulation with palmitic acid or linoleic acid did not change messenger RNA (mRNA) expression of OPG (*n* = 3) and RANKL (*n* = 1) by RA osteoblasts, which could be confirmed for RA and OA osteoblasts by protein quantification via ELISA. sRANKL remained below detection level (<370 pg/ml) in both RA (*n* = 3) and OA osteoblasts (*n* = 3), while OPG was detectable but showed no changes (RA OB 1.2-fold change, *p* > 0.05/OA OB 1.0-fold change, *p* > 0.05; *n* = 3 each).

### Effect of Free Fatty Acids on Expression of Central Wnt Signaling Molecules

As the Wnt signaling pathway is one of the pathways that affect osteoblast differentiation and activity, we analyzed the mRNA expression of two of its key molecules: axin-2 (conductin) and β-catenin. However, neither palmitic acid nor linoleic acid changed the mRNA expression in RA (1.1-fold change, *p* > 0.05; *n* = 3) or OA osteoblasts (1.5-fold change, *p* > 0.05; *n* = 3).

### Analysis of the Role of TLR2 and TLR4 in Palmitic-Acid-Induced IL-8 Secretion by Osteoblasts

**Figure 3:**
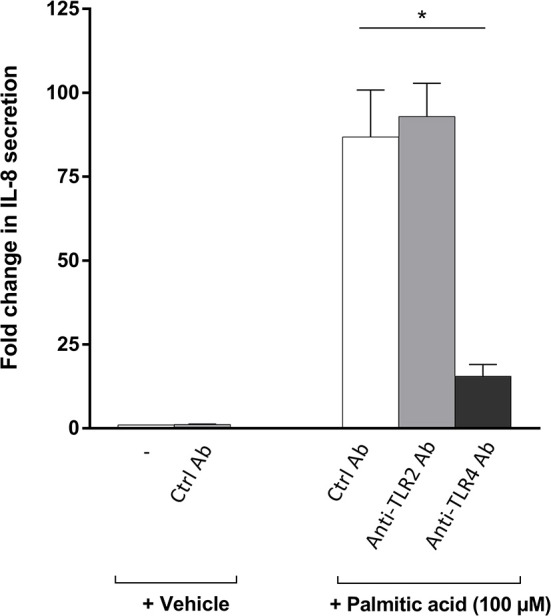
Blocking Toll-like receptor (TLR) 4 but not TLR2 significantly reduces the palmitic-acid-mediated interleukin-8 (IL-8) secretion by osteoblasts. TLR2 and TLR4, two receptors for free fatty acid (FFA), were blocked by neutralizing antibodies on rheumatoid arthritis (RA) osteoblasts (*n* = 3). Palmitic-acid-induced IL-8 secretion was significantly reduced by the anti-TLR4 antibody but not by the anti-TLR2 antibody or the isotype control antibody. *p < 0.05. Ctrl, control; Ab, antibody; TLR, Toll-like receptor.

TLR2 and TLR4 have previously been described as receptors for FFA (15, 44–47). In addition, previous results suggest that TLR4 may be involved in arthritis-dependent joint destruction (32, 48, 49). To analyze the role of these TLRs in fatty-acid-mediated effects on RA osteoblasts, we specifically blocked TLR2 or TLR4 using neutralizing antibodies and quantifying osteoblast-secreted IL-8, the chemokine which showed the highest induction by palmitic acid and linoleic acid. In these experiments, palmitic acid was used as the stimulant. The neutralizing TLR4 antibody significantly reduced the IL-8 secretion, while the neutralizing TLR2 antibody had no effect on the RA osteoblasts (Figure 3, *n* = 3 each).

### Effect of Free Fatty Acids on Mineralization Activity of Osteoblasts

**Figure 4:**
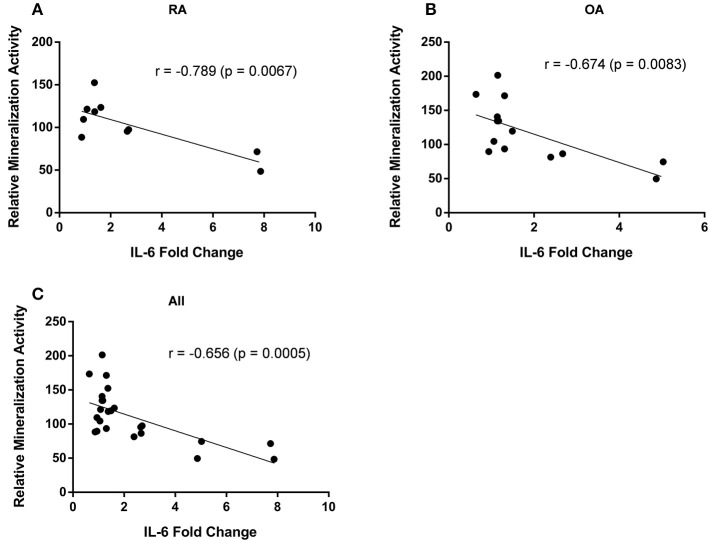
Mineralization activity of osteoblasts correlates with their IL-6 secretion. The mineralization activity of rheumatoid arthritis (RA) (*n* = 10) and osteoarthritis (OA) (*n* = 14) osteoblasts was inversely correlated with the changes in IL-6 secretion levels. There was a significant inverse correlation for RA **(A)** and OA osteoblasts **(B)** as well as the combination of both **(C)**.

Mineralization activity of osteoblasts varied between different cell populations (i.e., cells from different patients) as did the changes in IL-6 secretion induced by FFA. We therefore analyzed whether the osteoblastic mineralization activity correlated with the changes in IL-6 secretion. A significant inverse correlation could be found between these parameters for RA (*r* = −0.79, *p* = 0.0067; *n* = 10) and OA osteoblasts (*r* = −0.67, *p* = 0.0083; *n* = 14) as well as the combination of both (*r* = −0.66, *p* = 0.0005; *n* = 24), i.e., the mineralization activity was reduced with increasing IL-6 secretion levels (Figure 4).

### Influence of Free Fatty Acids on Osteoclasts

Preliminary experiments with murine osteoclasts (differentiated from bone marrow cells) showed an increased secretion of IL-6, macrophage inflammatory protein (MIP) 1,α and MIP-1β (*n* = 3) at day 4 of differentiation when treated with FFA for 3 days (Figure S2). Of note, murine osteoclast precursors differentiate more quickly than human osteoclast precursors.

**Figure 5:**
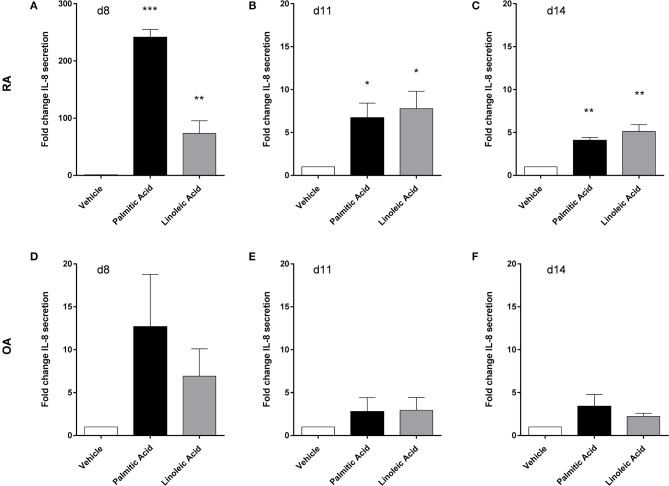
Human osteoclasts and their precursors from rheumatoid arthritis (RA) patients respond differently to free fatty acids than their counterparts from osteoarthritis (OA) patients. Palmitic acid and linoleic acid significantly increased the interleukin-8 (IL-8) secretion of RA osteoclasts and their precursors (*n* = 4), particularly at the earliest time point (d8) **(A–C)**, while equivalent cells from OA patients (*n* = 4) only showed weaker effects without statistical significance **(D–F)**. *p < 0.05; **p < 0.001; and ***p < 0.001.

Human osteoclasts were differentiated from PBMC. At the final differentiation time point (d14), cathepsin K, CLCN7 (chloride channel 7), and TCIRG (subunit of a V-type proton ATPase), three markers of osteoclast activity, were not affected in RA osteoclasts (*n* = 3) by palmitic acid or linoleic acid at mRNA level. The FFA also had no effect on the mRNA expression of osteoclast-associated receptor and nuclear factor of activated T cells, cytoplasmic 1, two markers of osteoclast differentiation, at d14. MMP-9 secretion was not significantly changed by FFA at any of the analyzed time points (d8, d11, d140 (*n* = 4). FFA, however, changed the IL-8 secretion of osteoclasts and their precursors (*n* = 4) (Figure 5). This effect was most prominent at the earliest time point (d8) and weaker at the later time points (d11, d14). The changes did not reach statistical significance for OA cells (Figures 5D–F). Interestingly, there was a very big difference in the response of RA cells and OA cells at d8 [Figure 5A (fold change scale, 0–300)/Figure 5D (fold change scale, 0–20)], which was much less pronounced at the later time points.

## Discussion

High-fat diets have proven to be detrimental in the context of various mouse models of arthritis (27–29, 50). Although this type of diet generally results in obesity, the accompanying increased mechanical load does not appear to be the only or major factor leading to the aggravation of OA as shown by Griffin et al. (27). In addition, several observations in humans suggest that other factors are involved in the effects of obesity as has been elucidated in the introduction. These factors may include FFA. Furthermore, data from human studies as well as data from animal models suggest that lipid levels also affect bone remodeling (51–54). Dietary intake of saturated fats was inversely correlated with bone density in humans (55), suggesting a detrimental effect of these fats and their components on bone metabolism. As these factors are generally associated with increased FFA levels and as obesity has been found to be a risk factor for OA and RA (17–19), we hypothesized that chronically elevated FFA levels may have a negative impact on subchondral bone integrity in these rheumatic diseases.

It has previously been shown that saturated fatty acids including palmitic acid (56, 57) enhance RANKL-induced osteoclastogenesis. On the other hand, unsaturated omega-3 fatty acids such as docosahexaenoic acid and eicosapentaenoic acid decreased RANKL-induced osteoclastogenesis (58–60). However, the unsaturated omega-6 fatty acid linoleic acid did not have a direct effect on osteoclastogenesis (58, 59) and also did not affect bone resorption *in vitro* (59) in contrast to palmitic acid, which increased bone resorption on dentin disks (57). In our experiments, the stronger induction of IL-8 secretion by palmitic and linoleic acid in RA vs. OA osteoclasts indicates an increased sensitivity of the RA cells toward these FFA. In contrast, we did not observe such a difference between RA osteoblasts and OA osteoblasts but found similar inductions of several proinflammatory factors including the cytokine IL-6. Interestingly, the degree to which IL-6 secretion could be induced in the osteoblasts by FFA was inversely correlated with their relative mineralization activity, indicating a link between inflammation and reduced bone mineralization (61). The chemokine GRO-α was not induced across all osteoblast populations examined but only in a subset. Age, sex, and medication were not associated with GRO-α induction or the lack thereof, meaning that other patient characteristics must be responsible for the difference. These might be differences at the molecular level such as different receptor densities or expression levels of signaling molecules. Axin-2 and β-catenin, two key molecules of the Wnt signaling pathway (62), were not influenced by palmitic or linoleic acid in osteoblastic cells, suggesting that these fatty acids do not directly affect osteoblastogenesis. Although both TLR2 and TLR4 have previously been described as receptors for FFA in other cell types (15, 44–47), our results suggest a role of TLR4 but not TLR2 in palmitic-acid-induced effects on osteoblasts. This could partially explain the so-called “Adonis” phenotype of mice with a defective TRL4 receptor. In addition, TLR4-dependent pathways have been shown to be involved in joint remodeling in arthritis mouse models (48, 49). Besides low adiposity, this mouse phenotype is characterized by stronger bones with increased bone mineral content and density (63). This is also in agreement with the results by Oh et al. (56), which showed that the knockout of TLR4 eliminated the enhanced survival effect of saturated fatty acids on murine osteoclasts. Not all FFA are equal in structure and effect. There are short-, medium, and long-chain fatty acids as well as saturated and unsaturated fatty acids. In the context of rheumatic diseases, omega-3 fatty acids have already been analyzed in animal studies and clinical trials. Omega-3 fatty acids belong to the group of polyunsaturated long-chain fatty acids with a specific location of their first double bond within the carbon backbone, the ω-3 position. These fatty acids are generally considered anti-inflammatory as they are precursors to anti-inflammatory molecules such as prostaglandin E3, leukotriene B5, resolvins, or protectins, and may exert anti-inflammatory effects via other mechanisms including disruption of lipid rafts, inhibition of proinflammatory, and activation of anti-inflammatory transcription factors (64). Clinical trials have shown some benefits of omega-3 fatty acids for RA patients (64), mostly when used in conjunction or as a supplement to conventional therapeutics. Bone loss in a post-menopausal mouse model (mediated by ovariectomy) could be alleviated by generation of Fat-1 transgenic mice. The Fat-1 transgene confers the capability of synthesizing omega-3 fatty acids from omega-6 fatty acids, thus leading to increased omega-3 fatty acid levels *in vivo* (65). Knocking out the enzyme delta-5 desaturase (D5D), which is involved in the synthesis of several polyunsaturated fatty acids including the omega-6 fatty acid linoleic acid, improved the metabolic profile of mice and reduced the inflammation in a vascular injury model (66). Hence, inhibitors of D5D might prove useful in ameliorating FFA-related joint/bone damage. Whether statins as lipid-lowering drugs provide beneficial effects in RA (67, 68) and/or OA (69, 70) is discussed controversially, and subchondral bone degradation has not been part of the investigations. Transdermal drugs that can be applied topically to affected joints might be an alternative approach as Gutierrez et al. (53) could show enhanced bone fracture repair in rats when treated with the transdermal statin lovastatin.

In conclusion, we could identify a new potential mechanism by which adipose tissue might contribute to subchondral bone damage and specifically inflammation-driven bone destruction in RA and OA.

## Data Availability Statement

All datasets generated for this study are included in the article/Supplementary Material.

## Ethics Statement

The studies involving human participants were reviewed and approved by Ethics Committee of the Justus-Liebig-University of Giessen. The patients/participants provided their written informed consent to participate in this study. The animal study was reviewed and approved by local ethics authorities of the Regierung of Unterfranken.

## Author Contributions

All authors listed have made a substantial, direct and intellectual contribution to the work, and approved it for publication.

### Conflict of Interest

The authors declare that the research was conducted in the absence of any commercial or financial relationships that could be construed as a potential conflict of interest.
