# Supplementary figures and images for: Free Fatty Acids in Bone Pathophysiology of Rheumatic Diseases
Source: Front Immunol. 2019 Dec 3;10:2757. doi: 10.3389/fimmu.2019.02757 (PMC6901602; doi:10.3389/fimmu.2019.02757)

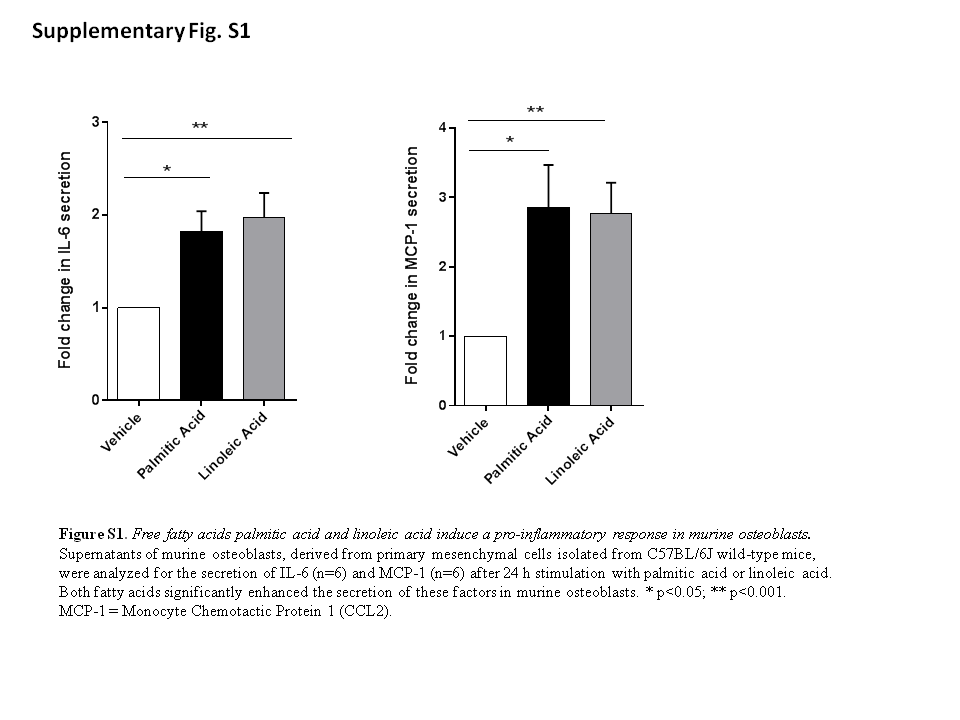

Supplement: Supplementary file 1 [file Image_1.TIF]

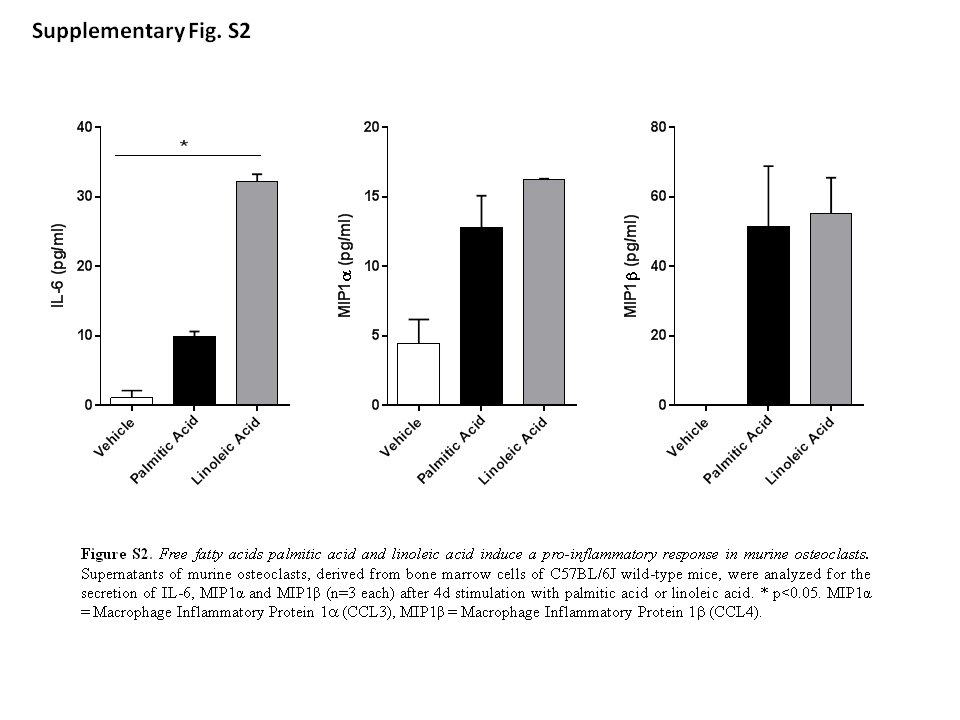

Supplement: Supplementary file 2 [file Image_2.TIF]
